# In memoriam: John R. Froines, PhD

**DOI:** 10.1002/ajim.23421

**Published:** 2022-08-09

**Authors:** Philip J. Landrigan

**Affiliations:** ^1^ Global Public Health Program Boston College Chestnut Hill Massachusetts USA


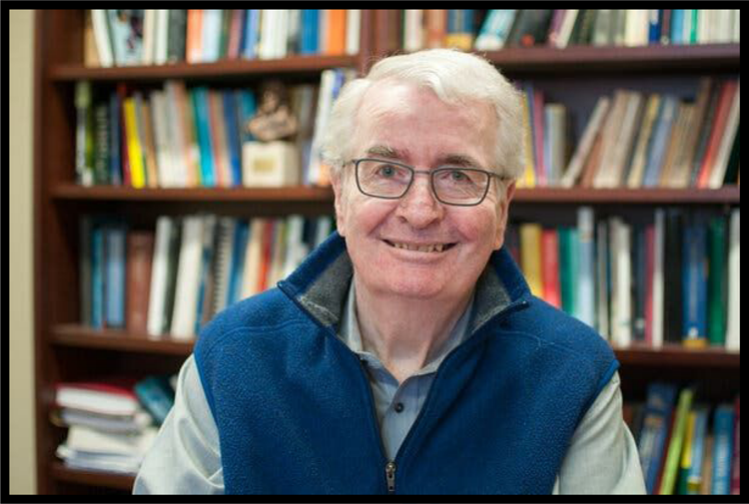


To the Editor,

John Froines is best known as a member of the famed Chicago Seven, the group accused of conspiring to incite a riot during the 1968 Democratic National Convention. That aspect of John's life is brilliantly recounted in his *New York Times* obituary of July 14, 2022.^1^

Less widely known, but also of great importance is John Froines' work as a leading toxicologist who made lasting contributions to occupational and environmental health. John's work in this field combined his passions for social justice and science and built on his training as a chemist. He received a PhD in physical chemistry at Yale in the 1960s, in the laboratory of Professor Kenneth Wiberg.

John's first position in occupational and environmental health was his appointment as Director of the Vermont Occupational Safety and Health Program. He played a key role there in developing effective regulatory standards for Vermont Yankee, the state's now defunct nuclear power plant.

After 3 years in Vermont, Froines moved to Washington, DC to become head of toxic chemical standards for the Occupational Safety and Health Administration, then headed by Dr. Eula Bingham in the Presidential administration of Jimmy Carter. He immediately sought to address one of the most significant worker health issues of the century by leading the development of a landmark standard to prevent workers against lead poisoning in the hundreds of industries still using lead in the 1970s. Two unique elements of this standard were its inclusion of requirements for medical removal protection and rate retention for workers mandatorily removed from high‐paying production jobs because of elevations in their blood lead levels. Froines also made major contributions to the cotton dust exposure standard designed to protect workers against byssinosis. This standard was overturned by the Reagan administration in the first weeks after they came into office in January 1981.

From OSHA, John went on to serve as the Deputy Director of the National Institute for Occupational Safety and Health (NIOSH) under Dr. Anthony Robbins. There he oversaw great expansion of the Health Hazard Evaluation program that enabled NIOSH investigators to enter workplaces to assess occupational hazards at the request of exposed workers and their unions. Additionally, he launched a major reassessment of the carcinogenicity of benzene, which came to the unequivocal conclusion that occupational exposure to benzene causes leukemia and lymphoma at even the lowest concentrations.

In 1980, Froines moved to the University of California, Los Angeles (UCLA) where he became a professor of toxicology and served in that position for more than 30 years. There he directed numerous occupational and environmental scientific centers funded by the US EPA and the State of California. He was a prominent member of the California Air Resources Board (CARB) and made seminal contributions to protecting public health in California against toxic and carcinogenic diesel exhaust emissions.

Froines was the recipient of many awards. In 2011, the California Air Resources Board honored John as an “outstanding individual who has made significant contributions toward improving air quality throughout a lifetime of commitment and leadership and innovation in research and environmental policy.”

Physicians for Social Responsibility, Los Angeles, recognized Froines in 2012 for his “courageous commitment to scientific integrity and for increasing our understanding of the health impacts of toxic chemicals on the health of workers and communities.”^2^ Author and Occidental College Emeritus Professor Robert Gottlieb presented the award to Froines, stating: “He has turned science into policy… while also being harassed and challenged by industry polluters—including the chemical companies and the pesticide manufacturers.”

In 2013, Froines was awarded the Ramazzini Award by the Collegium Ramazzini, an international society of leaders in occupational and environmental health based in Italy. This award recognized Froines for “his outstanding career in occupational and environmental health research and advocacy, especially his pioneering work to help develop the federal occupational lead and cotton dust exposure standards in the United States and his work in California that led to the recognition of diesel exhaust as a toxic air contaminant, preserving the health and the lives of millions.”^3^, ^4^

Froines was recognized by environmental justice activists, including East Yard Communities for Environmental Justice which honored him in 2015. Angelo Logan, Los Angeles activist and cofounder of the organization, credits Froines for “bringing science to the people impacted by pollution” and for his “landmark work in creating a regulation for reducing diesel exposure, a significant problem in lower‐income communities of color” often surrounded by truck and ship exhaust.

John Radford Froines was born on June 13, 1939, in Oakland, CA. He graduated from Berkeley High School, the University of California at Berkeley and obtained a PhD from Yale University. His parents were shipyard workers in Oakland during World War II, and his father was tragically murdered while returning home from the shipyards when John was only 3 years old.

John was a lifelong athlete; he was a star football player at Berkeley High School, where he was elected to the “Hall of Fame,” ran more than 25 marathons, and excelled at expert slopes when skiing in Steamboat Springs, CO, where he went at least once a year from his 40's to his 70's.

Froines is survived by his wife of 42 years, Andrea Hricko, a professor emerita in environmental health at the University of Southern California Keck School of Medicine; his daughter Rebecca Froines Stanley, director of nursing, psychiatry, and behavioral health and psychiatric emergency services at Yale New Haven Hospital; his son Jonathan Froines, a horticulturalist and garden designer, and numerous nieces and nephews.

## CONFLICT OF INTEREST

The author declares no conflict of interest.

## DISCLOSURE BY AJIM EDITOR OF RECORD

John Meyer declares that he has no conflict of interest in the review and publication decision regarding this article.

## AUTHOR CONTRIBUTIONS

Philip J. Landrigan is the sole author of this manuscript.
